# Vectored-Vaccine Platforms Enabled Rapid Development of Safe and Effective Vaccines in Response to COVID-19 Pandemic Situation

**DOI:** 10.3390/vaccines9070722

**Published:** 2021-07-02

**Authors:** Amine A. Kamen

**Affiliations:** Department of Bioengineering, McGill University, Montreal, QC H3A 0E9, Canada; amine.kamen@mcgill.ca

In 2019, an outbreak of severe acute respiratory syndrome coronavirus 2 (SARS-CoV-2), the causative agent of coronavirus disease (COVID-19), caused an ongoing public health crisis. 

In response, an unprecedented mobilization of public health authorities, the international scientific community, and industrial organizations was supported by major funding initiatives at the national and multinational level to develop a vaccine. Within a year, this led to the design, development, manufacturing, clinical evaluation, regulatory approval, and deployment of safe and efficacious vaccines against SARS-CoV-2. 

Genomic vaccines were shown to be highly successful in this application, with mRNA-based products the front-runners in the race to develop a vaccine against SARS-CoV-2. This has been acknowledged as a “technological jump” in the field of vaccinology, with numerous potential applications in other fields, including cell and gene therapy.

Vectored vaccines, more specifically adenovirus-vectored vaccines, rapidly followed in the approval process of safe, effective, and protective COVID-19 vaccines. These include the AstraZeneca-Oxford vaccine using recombinant chimpanzee adenovirus 2 (ChAdOx2), the Johnson & Johnson (Janssen) vaccine using recombinant human adenovirus 26, the CanSino biologics vaccine using the well-established recombinant human adenovirus 5 (Ad5), and the Gamaleya Institute’s Sputnik V vaccine using recombinant adenovirus 26 followed by Ad5 in the second heterologous boost (https://www.who.int/publications/m/item/draft-landscape-of-covid-19-candidate-vaccines, accessed on 2 July 2021).The hundreds of millions successfully vaccinated to date with vectored vaccines, as well as the associated phase 4 monitoring data, will further establish vectored vaccines as an important platform in the toolbox of modern vaccinology.

The content assembled in this Special Issue titled “vectored vaccines” captures some of the scientific and technological efforts that have been dedicated to advancing the field and contribute to better global preparedness for future emerging and re-emerging infectious diseases. The full-length spike (S) glycoprotein of SARS-CoV-2 has been rapidly identified as a target of neutralizing antibodies and a dominant antigen of SARS-CoV-2 based on previous work on coronaviruses such as SARS-Co-1 and Middle East respiratory syndrome (MERS).

Taking advantage of this prior knowledge, Farnós et al. [1] demonstrates the production of the receptor binding domain (RBD) of the spike protein of SARS-CoV-2 using the Ad5 expression system and the transient transfection technology in suspension culture to accelerate the development of adjuvanted subunit COVID-19 vaccines. Benest et al. [2] analyzed published data from clinical trials of Ad5-vectored COVID-19 vaccines to assess the minimal dose required for protection and maximum immunogenicity while maintaining safety. This provided data for a cost-effectiveness analysis to support the deployment of COVID-19 Ad-5 vaccines worldwide. Baldo et al. [3], based on the observation that recombinant viral vector vaccines represent a significant portion of vaccines that have either been approved or are under development, review the regulatory requirements for these products as genetically modified organisms (GMOs) from an environmental risk assessment (ERA) perspective. 

Heterologous prime-boost strategies are known to substantially increase immune responses in viral-vectored vaccines. Work in this area is also highly relevant to questions currently posed by public health authorities about the effectiveness of using different vaccines for the first and second dose. In this Special Issue, Folegatti et al. [4] report on the safety and immunogenicity of a poxvirus modified vaccinia Ankara (MVA) vaccine expressing four *Mycobacterium avium* subspecies *paratuberculosis* antigens, administered either as a single dose or as a booster vaccine following an initial dose of ChAdOx2. The study demonstrates that a heterologous prime-boost schedule is well tolerated and induced T-cell immune responses.

Other viral vectors than adenoviruses have been considered. Sun et al. [5] propose the use of Newcastle disease virus (NDV) expressing SARS-CoV-2 spike protein as an inactivated vaccine, taking advantage of existing large-scale egg-based vaccine production capacities. In a similar vein, Rohaim et al. [6] engineered an avirulent strain of avian orthoavulavirus 1 (AOaV-1) to express SARS-CoV-2 spike protein as a topical respiratory vaccine candidate against COVID-19.

The focus of the global research and development efforts on designing, developing, and delivering safe and effective COVID-19 vaccine candidates should not undermine the continuing efforts to develop vaccines against other infectious diseases. Choi et al. [7] explore the use of a recombinant baculovirus-vectored vaccine against Zika virus infection (ZIKV), demonstrating immunogenicity and protection in mice studies. The recombinant baculovirus vector is pseudo-typed with a human endogenous retrovirus envelope to enhance gene delivery of the premembrane and envelope genes of ZIKV, and the authors demonstrate immunogenicity and protection in mice studies. Additionally, this Special Issue details research efforts towards the development of a candidate vaccine against respiratory syncytial virus (RSV) by Sawada et al. [8]. Measles virus is another viral vector that retained attention in the development of COVID-19 vaccine candidates. The ectodomains of measles fusion and hemagglutinin proteins from measle virus (MV) were replaced with those of RSV F and G proteins, resulting in a chimeric MV/RSV vectored vaccine.

Global efforts to control infectious disease dissemination through the vaccination of animals emphasize the principle of “one-world, one-health”. Again, these principles have been highlighted by the continuing efforts to identify the origin of SARS-CoV-2 and its first transmission to humans. The bovine respiratory disease complex (BRDC) remains a major problem for both beef and dairy cattle industries worldwide and can compromise the livelihoods of many in low-income countries. To control BRDC, Chowdhury et al. [9] propose an improved quadruple gene mutated vaccine vector (BoHV-1 QMV), which expresses BVDV type 2, chimeric E2, and Flag-tagged Erns fused with bovine granulocyte monocyte colony-stimulating factor (GM-CSF). 

In conclusion, the recent development of adenovirus-vectored COVID-19 vaccines has demonstrated the safety and efficacy of vectored vaccines on a massive scale. To meet the needs of low- and medium-income countries and the control of COVID-19 globally, more efforts should be directed to improve the robustness of the manufacturing technologies and their cost-effectiveness.

## Data Availability

Not applicable.
